# Effect of Bidispersity on Dynamics of Confined Polymer Films

**DOI:** 10.3390/polym10121327

**Published:** 2018-11-30

**Authors:** Sijia Li, Qiaoyue Chen, Mingming Ding, Tongfei Shi

**Affiliations:** 1 Department of Fire Command, China People’s Police University, Langfang 065000, China; lisijia_wjxy@126.com; 2 Xinjiang Laboratory of Phase Transitions and Microstructures in Condensed Matter Physics, College of Physical Science and Technology, Yili Normal University, Yining 835000, China; chenqy945@126.com; 3 State Key Laboratory of Polymer Physics and Chemistry, Changchun Institute of Applied Chemistry, Chinese Academy of Science, Changchun 130022, China; 4 School of Applied Chemistry and Engineering, University of Science and Technology of China, Hefei 230026, China

**Keywords:** bidispersity, dynamics, polymer films

## Abstract

Using Monte Carlo simulations, we studied the effect of bidispersity on the dynamics of polymer films capped between two neutral walls, where we chose three representative compositions for bidispersed polymer films. Our results demonstrate that the characteristic entanglement length is an important parameter to clarify the effect of the bidispersity on the dynamics of polymer films. For the short chains, shorter than the characteristic entanglement length, the average number of near-neighboring particles increases with the decrease of the film thickness and limits the diffusivity of the short chains, which is independent of the film compositions. However, the dynamics of the long chains, of which is above the characteristic entanglement length, is determined by the film’s composition. In our previous paper, we inferred from the structures and entanglements of the bidisperse system with short and long chains that the constraint release contributes significantly to the relaxation mechanism of long chains. By calculating the self-diffusion coefficient of long chains, we confirmed this prediction that, with a lower weight fraction of long chains, the self-diffusion coefficient of long chains decreases slowly with the decrease of the film thickness, which is similar to that of short chains. With a higher weight fraction of long chains, the competition between the disentanglement and the increased in the local degree of confinement which resulted in the self-diffusion coefficient of long chains varying non-monotonically with the film thickness. Furthermore, for the bidisperse system with long and long chains, the diffusivity of long chains was not affected by the constraint release, which varied nonmonotonically with the decrease of the film thickness due to the competition between the disentanglement and the enhanced confinement. Herein, compared with the previous work, we completely clarified the relationship between the structures and dynamics for three representative compositions of bidisperse polymer films, which contains all possible cases for bidisperse systems. Our work not only establishes a unified understanding of the dependency of dynamics on the bidispersity of polymer films, but also helps to understand the case of polydispersity, which can provide computational supports for various applications for polymer films.

## 1. Introduction

The dynamic properties of thin polymer films are the key physical factors for their widespread technological applications such as surface coatings, adhesives, lubricants, composite materials, etc. [1,2,3,4,5,6], and therefore have received intense interests in the past decades. However, much of this attention has focused on elucidating the changes in the monodisperse polymer films under different types of confinement [7,8,9,10,11,12,13,14,15,16,17]. The fundamental role that the inevitable existence of polydispersity in polymer films plays in their dynamic properties has not yet been well-understood.

Polydispersity is known to affect the structural and thermodynamic properties [18,19] and even the processing of polymer melts [20,21]. The tube model has given a specific description of the effects of polydispersity on the dynamics of bulk systems [22]. For monodisperse polymer melts, the relaxation time of the surrounding chains determines the lifetime of an entanglement that confines a chain inside a tube, which means that chains crawl out the tube like a slithering snake, known as “reptation” [22]. In contrast, for polydisperse polymer melts, due to the fast relaxation of short chains, the entanglements formed by short and long chains can quickly dissolve, which results in the lifetime dependence of the entanglements on the involved chain length [23]. As a consequence, because of the possible motion in a direction perpendicular to its contour line [24], the long chains relax more quickly than that of a pure reptation process [23].

However, a unified understanding of the dependency of the dynamic properties of polymer films on polydispersity has not been achieved. The experiments investigating the effects of bidispersity and tridispersity on a wall slip of polymer films have indicated that short chains can enhance the slip of entangled chains on non-wetting surfaces, which may come from the effects of chain end groups or short chain enrichment on the effective friction coefficient [25,26,27]. In fact, simulation studies have also revealed a significant slip for short chains [28], which indicates that the simulations provide an excellent tool to obtain a molecular scale understanding. The dynamic Monte Carlo study has found that polydispersity can reduce the confinement effects on the orientation and relaxation time of polymer chains, where the polydispersity can provide more degrees of freedom for chain configurations [29], In our previous work, we have shown that short chains can significantly affect the local situations of entanglements, and predicted the relaxation mechanisms of long chains based on constraint release dynamics [30], Actually, the entanglement plays a key role in the dynamics properties of polymers. Nevertheless, a link between the changes of entanglements and the changes of dynamics of polymer films induced by polydispersity has hardly been explicitly established in previous studies.

In this work, using Monte Carlo simulations, we constructed the simplest case of polydispersity, a bidisperse mixture, to investigate the effect of polydispersity on the dynamics of confined polymer films, which can represent the dynamics of polymer chains in bulk melts as well as in confined cases [17,31,32,33]. We considered three model systems in our simulations: The first model system consisted of two short chains of which the molecular lengths were both below the characteristic entanglement length; the second one consisted of short chains of which the molecular length was below the characteristic entanglement length, and long chains of which the molecular length was above the characteristic entanglement length; the third one consisted of two long chains of which the molecular lengths were both above the characteristic entanglement length. In our previous work, we only calculated the structures and entanglements of the second model system [30]. However, in this work, we chose three representative compositions of bidisperse polymer films (containing all possible cases for bidisperse systems), thus we could completely clarify the relationship between the structures and dynamics for bidisperse polymer films. The degree of entanglement of polymers was extracted by a geometric analysis method (*Z*1) [34,35,36,37], which has been proven to be suitable for analyzing anisotropic samples [13,14,38], The remainder of this article is organized as follows: In Simulation Methods, we described the simulation methods and the corresponding simulation details. In Results and Discussion, we systematically investigated the effect of bidispersity on the dynamics of short and long polymers. Our simulations revealed the molecular mechanisms of the effect of bidispersity on the dynamics of the polymers in these confined systems and among which differences depend on the film composition. In Conclusions, we briefly summarized our results and provided some concluding remarks.

## 2. Model and Simulation Method

We constructed a coarse-grained computational model for polymer melts capped between two hard walls and performed the simulations using the lattice-based Monte Carlo model introduced by Shaffer (S-BFM); interested readers could read our previous papers and other references for more details [17,30,31,32,33]. In our model, each monomer on the polymer chains occupied a single site on the primary cubic lattice with a lattice constant *a*_p_ = 1, and the bond lengths were allowed to fluctuate with the values of 1, 2^1/2^, and 3^1/2^. Polymer configurations could evolve through local displacements of one single monomer by forbidding double occupancy of the primary lattice sites and maintaining chain connectivity.

In this work, lengths are given in the unit of primary lattice spacing (*a*_p_), and times are given in the unit of attempted Monte Carlo steps (MCs) per monomer. We have demonstrated that the critical entanglement length from the unentangled to the entangled region in the bulk is N_e_ ≈ 33 by the Z1 algorithm in our previous study [17], which is agreement with previous works [31,32,39]. Hence, we chose the length of *N*_s_ = 10 and *N*_l_ = 20 for the first model system, *N*_s_ = 20 and *N*_l_ = 150 for the second one, and *N*_s_ = 150 and *N*_l_ = 300 for the third one, to cover both the unentangled and entangled regions for chains. We defined the weight fraction of the longer chains in the system as *φ*_l_ = *N*_l_*n*_l_/(*N*_s_*n*_s_ + *N*_l_*n*_l_), where *n*_s_ and *n*_l_ are the numbers of the shorter and longer chains, respectively. We set *φ*_l_ as 16.7%, 33.3%, 50.0%, 66.7% and 83.3% (see Table 1 for details).

We put both the shorter and longer chains into a rectangular simulation box with dimensions of (*L*_x_, *L*_y_, *L*_z_), where periodic boundary conditions were applied in the x and y directions. Moreover, we fixed two flat and impenetrable walls at *z* = 0 and *L*_z_ + 1 in the z direction, which were constructed of stationary particles (each particle occupies a single lattice site). Thus, we could consider the neutral walls by the excluded volume interaction between the monomers and the wall particles. In all cases, we set the fraction of occupied lattice sites as one-half, which has been shown to represent polymer melts in this model [39]. We adjusted the film thickness from 2 to 54 in our simulations (see Table 2 for details).

**Figure 1:**
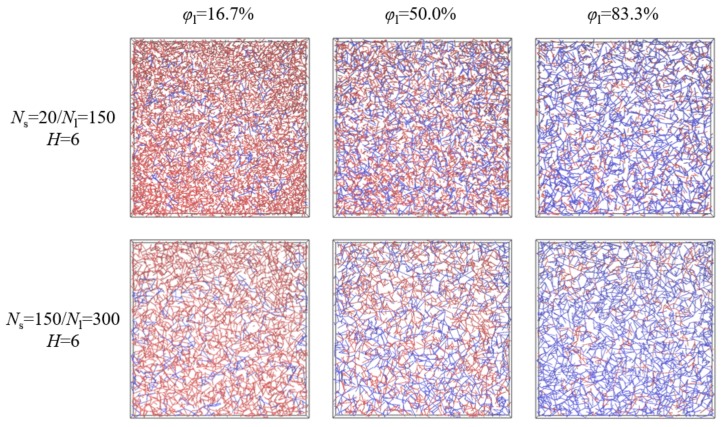
Representative primitive path networks with the shorter (**red**) and longer (**blue**) chains for polymer films of *N*_s_ = 20/*N*_l_ = 150 and *N*_s_ = 150/*N*_l_ = 300, where the film thickness is fixed as *H* = 6 and the weight fraction of the longer chains is changed from *φ*_l_ = 16.7% to *φ*_l_ = 83.3%.

First, we put both the shorter and longer chains on the lattice and run 10^6^–10^8^ MCs to equilibrate the system, where the chain crossing is allowed. Then, 10^6^–10^8^ MCs are run to gradually remove those crossings. Finally, we further ran 10^7^–10^8^ MCs to obtain the equilibrium configurations. For each composition of the model system, 10 parallel simulations with different random seeds were performed to average the results. The Z1 algorithm uses geometrical moves to monotonically reduce the chain contour lengths to the limit of infinitely thin primitive path (PP) thickness, which is suitable to analyze chain entanglements in the equilibrium configurations [34,35,36,37]. As shown in Figure 1, for polymer films of *N*_s_ = 20/*N*_l_ = 150, the density of entanglements formed among long chains played a key role in the confinement of the long-chain mobility, which evidently increases with the increase of the weight fraction of the longer chains for the same film thickness. In contrast, for polymer films of *N*_s_ = 150/*N*_l_ = 300, since the molecular length of the shorter chains is above the characteristic entanglement length, entanglements formed among shorter and longer chains all had a great effect on the chain mobility, and the total density of entanglements hardly varied with the weight fraction for the same film thickness. As discussed in our previous paper, we could obtain the diffusion coefficient by extrapolation in the limit of large simulation time [17]. Although this apparent diffusion coefficient is different from a true diffusion constant obtained for a sufficiently long time, we could use this apparent diffusion coefficient as a measure of dynamic difference, which was valid for both Rouse and reptation regions [30].

In order to compare the un-constrained monodisperse polymer melts, we also simulated the cases in the absence of walls (periodic boundary conditions are applied in all three directions). The simulation details and corresponding results are shown in Table 3.

## 3. Results and Discussion

### 3.1. Model System of N_s_ = 10/N_l_ = 20

**Figure 2:**
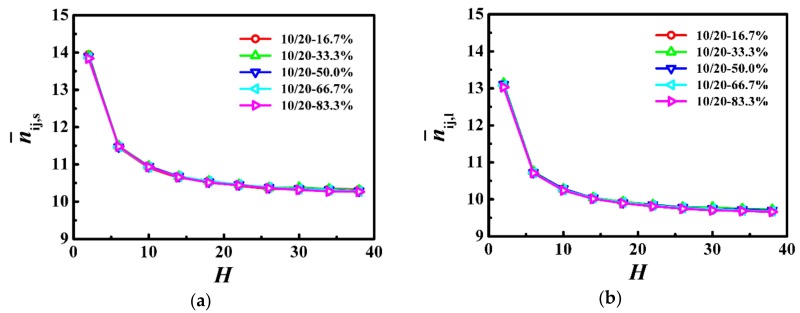
Average number of near-neighboring particles per monomer for (**a**) shorter (${\bar{n}}_{ij,s}$ ) and (**b**) longer (${\bar{n}}_{ij,l}$) chains as a function of the film thickness (*H*) in the model system of *N*_s_ = 10 and *N*_l_ = 20.

Firstly, we used the parameter of the number of near-neighboring “particles” per monomer (${\bar{n}}_{ij}$) to evaluate the local degree of confinement, where i is the monomer of a given chain and *j* includes monomers from other chains as well as wall particles within the distance of 3^1/2^ on the primary cubic lattice [17,30]. Figure 2 shows ${\bar{n}}_{ij}$ for the shorter and longer chains as a function of the film thickness, respectively. ${\bar{n}}_{ij}$ shows the similar trend, that is it is the largest for the thinnest film, which decreased rapidly as the film thickness was increased, and finally became a slowly decreasing function of the film thickness, which indicates that the local degree of confinement is independent of the weight fraction in the bidisperse polymer films.

**Figure 3:**
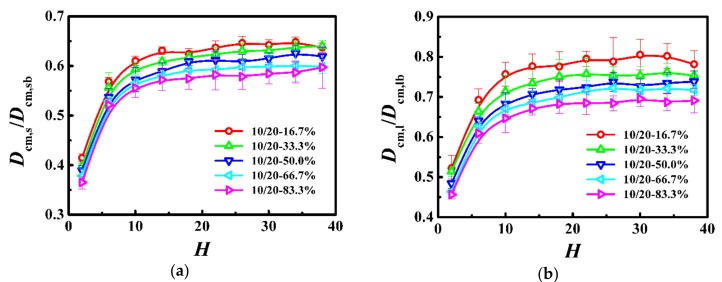
Normalized self-diffusion coefficient of the center-of-mass for (**a**) shorter (*D*_cm,s_/*D*_cm,sb_) and (**b**) longer (*D*_cm,l_/*D*_cm,lb_) chains as a function of the film thickness (*H*) in the model system of *N*_s_ = 10 and *N*_l_ = 20.

To further investigate the effect of bidispersity on the dynamics of polymer films, in Figure 3, we respectively calculated the self-diffusion coefficient of the center-of-mass for the shorter (*D*_cm,s_) and longer (*D*_cm,l_) chains as a function of the film thickness, and normalized the diffusion coefficient by that in the unconstrained monodisperse melts (see *D*_cm,b_ in Table 3). For the shorter and longer chains, the diffusion coefficient is a slowly decreasing function with the decrease of the film thickness in thicker films, whereas it becomes a rapidly decreasing function with the decrease of the film thickness in thinner films. Moreover, our results indicate that the self-diffusion coefficient of the shorter and longer chains increased with the decrease of the weight fraction of the longer chains for the same film thickness. Besides, in bidisperse systems, the longer chains reside away from the walls and the shorter chains are close to the walls [30]. This spatial inhomogeneity may cause the diffusion coefficients of both components are always smaller than those of bulk systems. Thus, due to the fast relaxation of the shorter chains, the diffusion coefficient of the longer chains increases with the decrease of the weight fraction of the longer chains.

As *N*_s_ = 10 and *N*_l_ = 20 were both below the characteristic entanglement length (*N*_e_ ≈ 33), they formed few entanglements which hardly had any influence on their dynamics. With the slow increase of the average number of near-neighboring particles (see Figure 2), the self-diffusion coefficients of the shorter and longer chains decreased slowly with the decrease of the film thickness (see Figure 3). For the thinnest film, the average number of near-neighboring particles exhibited a rapid increase (see Figure 2), which resulted in the lowest diffusivity of the shorter and longer polymers (see Figure 3). Our results indicate that the local degree of confinement regulates the dynamics of polymers in bidisperse polymer films, where the shorter and longer chains are both in the unentangled region.

According to the Rouse theory [22,40], the self-diffusion coefficient of polymers depends on chain length as *D*_cm_ = *k*_B_*T*/(*ζN*) for monodisperse melts, where *ζ* represents the monomeric friction coefficient. However, the chain ends have a greater mobility than the central section of the chain, and the friction coefficient is proportional to chain length until the chains become sufficiently long that the enhanced mobility of the ends only plays a minor role in the overall diffusion [41,42,43]. Since the friction coefficient is a mean-field measure, for bidisperse polymer melts, the average friction coefficient can be generalized through a simple linear additivity of the overall friction coefficient and becomes *ζ*_B_ = *φ*_s_*ζ*_s_ + *φ*_l_*ζ*_l_, where *φ*_i_ is the weight fraction of polymer *N*_i_ [44]. The diffusion coefficient of the N_i_ polymer is *D*_cm,i_ =*k*_B_*T*/(*ζ*_B_*N*_i_). The value of *D*_cm_ calculated from this model agreed well with that measured from simulations [44]. In this model system, for the same film thickness, as the weight fraction of the longer chains was decreased, the weight fraction of the shorter chains was increased, and consequently the average friction coefficient of monomers exhibited a decrease which enhances the dynamics of the shorter and longer chains (see Figure 3), even though the local degrees of confinement remained rather unchanged (see Figure 2).

### 3.2. Model System of N_s_ = 20/N_l_ = 150

We have investigated the local degree of confinement for the model system *N*_s_ = 20 and *N*_l_ = 150 in our previous work [30], where the variations of the average number of near-neighboring particles were similar to those of the model system of *N*_s_ = 10 and *N*_l_ = 20 (see Figure 2). Although the disentanglement of the longer chains was in agreement with the monodisperse systems [13,14,45], we found that the number of short−long entanglements exhibited a dramatic increase whereas that of long−long entanglements exhibited a slight decrease with the decrease of the weight fraction of the longer chains.

**Figure 4:**
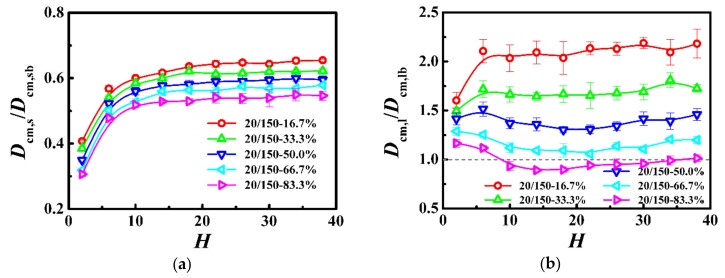
Normalized self-diffusion coefficient of the center-of-mass for (**a**) shorter (*D*_cm,s_/*D*_cm,sb_) and (**b**) longer (*D*_cm,l_/*D*_cm,lb_) chains as a function of film thickness (*H*) in the model system of *N*_s_ = 20 and *N*_l_ = 150.

To further investigate the effect of bidispersity on the dynamics of this model, we calculated the self-diffusion coefficient of the shorter (*D*_cm,s_) and longer (*D*_cm,l_) chains as a function of the film thickness in Figure 4, where the diffusion coefficient was normalized with respect to that in the unconstrained monodisperse melts (see *D*_cm,b_ in Table 3). For the shorter chains, the diffusion coefficient slowly decreased in thicker films and then rapidly decreased in thinner films with the decrease of the film thickness. In contrast, the variations of dynamics of the longer chains with the film thickness depend on the weight fraction. When the weight fraction of the longer chains is lower than one-half, the diffusion coefficient is a slowly decreasing function with the decrease of the film thickness in thicker films and becomes a rapidly decreasing function with the decrease of the film thickness in ultrathin films, which is similar to the variations of the dynamics of the shorter chains. However, if the weight fraction of the longer chains is not lower than one-half, the diffusion coefficient is a slowly decreasing function with the decrease of the film thickness, when the film thickness is greater than the bulk chain dimension (*H* > 2*R*_gl,b_), and an increasing function with the decrease of the film thickness when the film thickness is smaller than the bulk chain dimension (*H* < 2*R*_gl,b_). Moreover, when the weight fraction of the longer chains is equal to one-half, the diffusion coefficient in ultrathin films fluctuates slightly. Besides, the previous study has demonstrated that the diffusion coefficient of long chains is smaller than that of the bulk samples [46]. However, in this model system, compared to the long-long entanglements, the short-long entanglements dissolved quickly due to the fast relaxation of the shorter chains, which resulted in the diffusion coefficient of the longer chains greater than that of the bulk samples, especially for the lower weight fraction of the longer chains.

Because the molecular length of the longer chains was above the characteristic entanglement length (*N*_l_ = 150 > *N*_e_ ≈ 33), the shorter chains could form entanglements with them. However, those entanglements dissolved quickly due to the fast relaxation of the shorter chains and have an insignificant influence on their dynamics. As the film thickness was decreased, the self-diffusion coefficient of the shorter chains decreased slowly (see Figure 4) with a slow increase of the average number of near-neighboring particles (see Reference [30]). For the thinnest film, the rapid increase of the average number of near-neighboring particles (see Reference [30]) resulted in the lowest diffusivity of the shorter polymers (see Figure 4). Our results indicate that, the dynamics of the shorter chains in this model system was regulated by the local degree of confinement, which is similar to the dynamics of polymers in the unentangled bidisperse polymer films.

The Struglinski-Graessley parameter is defined as *G*_r_ = *N*_l_*N*_e_^2^/*N*_s_^3^, where *N*_l_ and *N*_s_ are respectively the lengths of the longer and shorter chains and N_e_ is the entanglement length. It can be interpreted as the ratio between the relaxation time of the longer chains due to the pure reptation and the relaxation time of the tube caused by constraint release [22]. For G_r_ larger than 1: The relaxation process is governed by the constraint release mechanisms [23,24,47]. The tube dilation model also predicted that, for higher weight fraction of the longer chains (*φ*_l_ > 1/*G*_r_), the number of long-long entanglements was high enough to restrict the longer chains within a tube even after the relaxation of the shorter chains. The decline of the entanglements causes the dilated tube diameter for the longer chains to increase to a certain extent, which is called “restricted tube dilation”. For the lower weight fraction of the longer chains (*φ*_l_ < 1/*G*_r_), however, the number of remaining long-long entanglements is not sufficient, so that the tube diameter can grow without limits and thus the process is called “free dilation” [23,24]. Previous molecular dynamics simulations have proved that the constraint release is the dominant relaxation mechanism in the melts of long and short chains [48,49]. In addition, a dissipative particle dynamics simulation has also provided strong evidence for the tube dilation of the long chains in bidisperse polymer melts [50], which supports the validity of the tube dilation model [23].

In this model system, the Struglinski-Graessley parameter was *G*_r_ = 20.4 > 1, which indicates that the constraint release mechanism plays a key role in the relaxation process of the longer chains. For the lower weight fraction of the longer chains, our results show that, as the average number of near-neighboring particles increased slowly (see Reference [30]), the self-diffusion coefficient of the longer chains decreased slowly with the decrease of the film thickness (see Figure 4); for the thinnest film, the average number of near-neighboring particles rapidly increased (see Reference [30]), which significantly limited the diffusivity of the longer polymers (see Figure 4). The change agrees with the prediction in our previous work [30], which is similar to that of the dynamics of the short chains. For the higher weight fraction of the longer chains, as the film thickness was decreased (*H* > 2*R*_gl,b_), the number of long-long entanglements remained unchanged (see Reference [30]), but the average number of near-neighboring particles slowly increased (see Reference [30]), which resulted in a slight reduction of the diffusivity of the longer polymers (see Figure 4). With a further decrease in the film thickness (*H* < 2*R*_gl,b_), the number of long-long entanglements rapidly decreased (see Reference [30]), which enhanced the dynamics of the longer polymers (see Figure 4). When the weight fraction of the longer chains was equal to one-half, in ultrathin films, the competition between the disentanglement and the increase in the local degree of confinement resulted in a slight fluctuation of the diffusion coefficient (see Figure 4).

### 3.3. Model System of N_s_ = 150/N_l_ = 300

**Figure 5:**
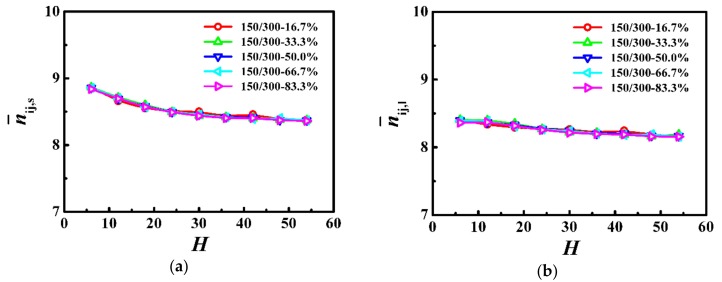
Average number of near-neighboring particles per monomer for (**a**) shorter (${\bar{n}}_{ij,s}$) and (**b**) longer (${\bar{n}}_{ij,l}$) chains as a function of the film thickness (H) in the model system of N_s_ = 150 and N_l_ = 300.

In Figure 5, we calculated the average number of near-neighboring particles per monomer as a function of the film thickness, which is a slowly decreasing function with the increase of the film thickness for both the shorter and longer chains. Moreover, the results from the average number of near-neighboring particles per monomer represent the local degrees of confinement for the shorter and longer chains, which are independent of the weight fraction in the polymer films.

**Figure 6:**
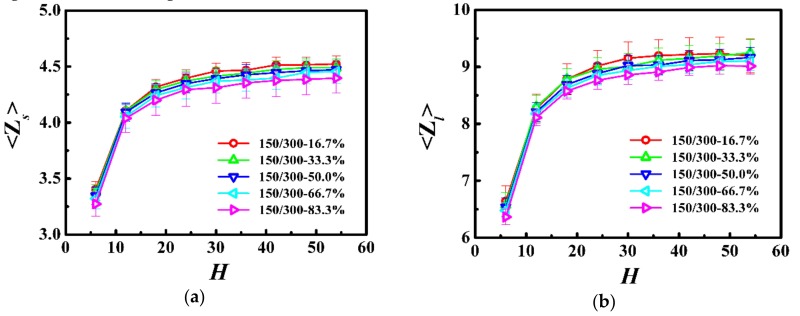
Average number of entanglements for each (**a**) shorter ($\left\langle Z_{s}\right\rangle$) and (**b**) longer ($\left\langle Z_{l}\right\rangle$) chain as a function of the film thickness (*H*) in the model system of *N*_s_ = 150 and *N*_l_ = 300.

In Figure 6, we respectively calculated the average number of entanglements for each shorter ($\left\langle Z_{s}\right\rangle$) and longer ($\left\langle Z_{l}\right\rangle$) chains in the PP network as a function of the film thickness. For the shorter and longer chains, the average number of entanglements was approximately independent of the film thickness in thicker films, however, it rapidly decreased with the decrease of the film thickness when the thickness was smaller than the coil size of the polymer (*H* < 2*R*_gs,b_ or H < 2*R*_gl,b_). In addition, our results indicate that the weight fraction in this model system had an insignificant influence on the degree of chain entanglements.

**Figure 7:**
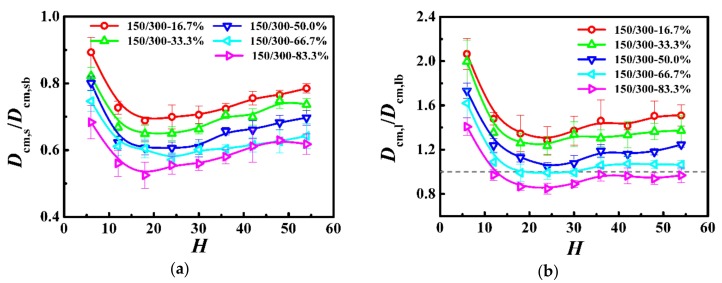
Normalized self-diffusion coefficient of the center-of-mass for (**a**) shorter (*D*_cm,s_/*D*_cm,sb_) and (**b**) longer (*D*_cm,l_/*D*_cm,lb_) chains as a function of the film thickness (*H*) in the model system of *N*_s_ = 150 and *N*_l_ = 300.

We further respectively calculated the self-diffusion coefficient of the center-of-mass for the shorter (*D*_cm,s_) and longer (*D*_cm,l_) chains as a function of the film thickness in Figure 7, and normalized the diffusion coefficient by that in the unconstrained monodisperse melts (see *D*_cm,b_ in Table 3). For the shorter and longer chains, the diffusion coefficient was a slowly decreasing function with the decrease of the film thickness when the thickness was greater than the bulk chain dimension (*H* > 2*R*_gs,b_ or *H* > 2*R*_gl,b_) and became a rapidly increasing function with the decrease of the film thickness when it was smaller than the bulk chain dimension (*H* < 2*R*_gs,b_ or *H* < 2*R*_gl,b_). Moreover, our results indicate that polymers exhibited higher diffusivity for the lower weight fraction of the longer chains in the polymer films.

In this model system, we fixed the lengths of the shorter and longer chains as *N*_s_ = 150 and *N*_l_ = 300, and the Struglinski-Graessley parameter was *G*_r_ = 0.1 < 1. The tube dilation model and the tube reptation model draw the same conclusions for polymer melts, with G_r_ smaller than 1, and agree that the chains reptated inside an undilated tube, and therefore, the relaxation of the longer chain component is not affected by constraint release [23,24,46]. The entanglements evidently impose a crucial effect on the dynamics of polymers in spite of the weight fraction in the polymer films. For the shorter and longer chains, as the film thickness was decreased (*H* > 2*R*_gs,b_ or *H* > 2*R*_gl,b_), the number of entanglements remained almost unchanged (see Figure 6), but the average number of near-neighboring particles slowly increased (see Figure 5), which results in a slight reduction of the diffusivity of the polymers (see Figure 7). With a further decrease in the film thickness (*H* < 2*R*_gs,b_ or H < 2*R*_gl,b_), the number of entanglements rapidly decreased (see Figure 6), which enhanced the dynamics of the polymers (see Figure 7).

## 4. Conclusions

In this work, we considered three model systems: First, when the molecular lengths of the shorter and longer chains were both below the characteristic entanglement length; second, when the molecular length of the shorter chains was below the characteristic entanglement length whereas that of the long chains was above it; third, when the molecular lengths of the shorter and longer chains were both above the characteristic entanglement length.

Our results demonstrate that, for short chains of which the molecular length is below the characteristic entanglement length, their dynamics is regulated by the local degree of confinement so that the diffusion coefficient decreases slowly in thicker films and then rapidly in thinner films with the decrease of the film thickness, which is independent of the composition of bidisperse polymer films. In contrast, the dynamics of long chains of which the molecular length was above the characteristic entanglement length depended on the film composition, which can be interpreted in terms of reptation and constraint release dynamics. For bidisperse film compositions with two long chains (Struglinsky-Graessley parameter G_r_ < 1.0), the dynamics of long chains was not affected by constraint release. Due to the competition between the decrease of the number of entanglements and the increase of the average number of near-neighboring particles, the diffusion coefficient varied nonmonotonically with the film thickness; the diffusion coefficient slowly decreased when the film thickness was greater than the bulk chain dimension (*H* > 2*R*_g,lb_) and rapidly increased when the film thickness was smaller (*H* < 2*R*_g,lb_) with the decrease of the film thickness. For bidisperse film compositions with short and long chains (*G*_r_ > 1.0), constraint release contributed significantly to the relaxation mechanism of the long chains. For lower weight fraction of long chains, the fast relaxation of short chains dilated the tube diameter of long chains, and consequently the local degree of confinement instead of the entanglements had a marked effect on the dynamics of long chains. As the film thickness was decreased, the increase of the average number of near-neighboring particles slowed down the diffusivity of long chains. However, for the higher weight fraction of long chains, after the relaxation of short chains, the long-long entanglements were in sufficient quantities to restrict the long chains within a tube, which implies that the dynamics of long chains is hardly affected by constraint release. The diffusion coefficient varied nonmonotonically with the decrease of the film thickness due to the competition between the disentanglement and the enhanced confinement. Compared with the previous work, we completely clarified the relationship between the structures and dynamics for all possible cases for bidisperse systems, which not only establishes a unified understanding of the dependency of dynamics on the bidispersity of polymer films, but also helps to understand cases of polydispersity.

## Figures and Tables

**Table 1:** Compositions of bidisperse polymer melts ^1^.

System (*N*_s_/*N*_l_-*φ*_l_)	*n*_s_	*n*_l_
10/20-16.7%	1120	112
10/20-33.3%	900	225
10/20-50.0%	674	337
10/20-66.7%	450	450
10/20-83.3%	224	562
20/150-16.7%	4500	120
20/150-33.3%	3600	240
20/150-50.0%	2700	360
20/150-66.7%	1800	480
20/150-83.3%	900	600
150/300-16.7%	960	96
150/300-33.3%	768	192
150/300-50.0%	576	288
150/300-66.7%	386	386
150/300-83.3%	192	480

^1^*n*_s_ and *n*_l_ are the numbers of the shorter and longer chains with the lengths *N*_s_ and *N*_l_, respectively, and *φ*_l_ represents the weight fraction of the longer chains in the system.

**Table 2:** Simulation details for bidisperse polymer films ^1^.

*N*_s_ = 10/*N*_l_ = 20			
*L*_x_ = *L*_y_	*H* = *L*_z_	*H*/*R*_gs,b_	*H*/*R*_gl,b_
116	2	1.04	0.71
68	6	3.11	2.14
52	10	5.18	3.57
44	14	7.26	4.99
38	18	9.33	6.42
36	22	11.40	7.85
32	26	13.47	9.27
30	30	15.54	10.68
28	34	17.62	12.10
26	38	19.69	13.52
***N*_s_ = 20/*N*_l_ = 150**			
***L*_x_ = *L*_y_**	***H* = *L*_z_**	***H*/*R*_gs,b_**	***H*/*R*_gl,b_**
330	2	0.71	0.25
190	6	2.14	0.74
146	10	3.57	1.24
124	14	4.99	1.74
110	18	6.42	2.23
100	22	7.85	2.73
90	26	9.27	3.23
84	30	10.68	3.72
80	34	12.10	4.22
76	38	13.52	4.72
***N*_s_ = 150/*N*_l_ = 300**			
***L*_x_ = *L*_y_**	***H* = *L*_z_**	***H*/*R*_gs,b_**	***H*/*R*_gl,b_**
240	6	0.74	0.52
170	12	1.49	1.04
138	18	2.23	1.56
120	24	2.98	2.08
106	30	3.72	2.60
98	36	4.47	3.12
90	42	5.21	3.64
84	48	5.96	4.16
80	54	6.70	4.68

^1^*L*_x_ = *L*_y_ is the lateral length of the simulation box, *H* = *L*_z_ denotes the film thickness, *H*/*R*_gs,b_ and *H*/*R*_gl,b_ represent the film thicknesses normalized by the mean-square radii of gyration of the shorter and longer chains in the unconstrained monodisperse melts, respectively.

**Table 3:** Simulation details and results for unconstrained monodisperse polymer melts ^1^.

*M*	*N*	*L*_x_ = *L*_y_ = *L*_z_	*R*_g,b_	*D*_cm,b_ × 10^6^	〈*Z*〉_b_
1350	10	30	1.93	1220.50	
675	20	30	2.81	444.43	
213	150	40	8.06	12.05	4.7162
208	300	50	11.53	2.24	9.2341

^1^ M represents the number of polymer chains, *N* denotes the length of polymer chains, *L*_x_ = *L*_y_ = *L*_z_ is the length of the simulation box, *R*_g,b_ denotes the mean-square radius of gyration of polymers, *D*_cm,b_ is the center-of-mass diffusion coefficient, and 〈*Z*〉_b_ represents the average number of entanglements per chain.
